# *“I always feel like I’m the first deaf person they have ever met:”* Deaf Awareness, Accessibility and Communication in the United Kingdom’s National Health Service (NHS): How can we do better?

**DOI:** 10.1371/journal.pone.0322850

**Published:** 2025-05-07

**Authors:** Bhavisha Parmar, Helen Henshaw, Shahad Howe, Ann Marie Dickinson, Crystal Rolfe, Philip Le Mere, Emmanuelle Blondiaux-Ding, Zara Musker, Rachel Stevenson, Sarah E. Hughes, Sian Calvert, Emma Stapleton, Laura Turton

**Affiliations:** 1 Sound Lab, Department of Clinical Neurosciences, University of Cambridge, United Kingdom; 2 British Society of Audiology, United Kingdom; 3 Ear Institute, University College London, United Kingdom; 4 NIHR Nottingham Biomedical Research Centre, Hearing Sciences, School of Medicine, University of Nottingham, United Kingdom; 5 Advanced Bionics, United Kingdom; 6 Salford Care Organisation, Northern Care Alliance, United Kingdom; 7 Royal National Institute for Deaf People (RNID), United Kingdom; 8 Leeds Dental Institute, National Deaf and Hard of Hearing (DHOH) NHS Staff Network, United Kingdom; 9 University of Manchester, United Kingdom; 10 Norfolk and Norwich University Hospital Trust,; 11 Centre for Patient Reported Outcome Research, University of Birmingham, Birmingham, United Kingdom; 12 Manchester Royal Infirmary, Manchester, United Kingdom; 13 NIHR Manchester Biomedical Research Centre, Manchester, United Kingdom; 14 NHS Tayside, Scotland, United Kingdom; Lamar University, UNITED STATES OF AMERICA

## Abstract

**Background:**

Barriers to communication significantly reduce access to health services for people with deafness or hearing loss (PDHL). These barriers contribute to reduced healthcare-seeking behaviour, poorer access to health information, and adverse health outcomes. In response, a multidisciplinary working group of patients, clinicians, researchers, and charity representatives was established to investigate accessibility, communication, and deaf awareness within the United Kingdom’s (UK) National Health Service (NHS).

**Methodology:**

A cross-sectional survey was conducted to explore the communication and accessibility experiences of PDHL NHS patients, and their perceived impact on well-being. The survey used rating scales and open-ended questions and data were analysed using descriptive statistics and thematic analysis. The survey was made available in British Sign Language (BSL).

**Results:**

The online survey was completed by 556 PDHL, including 50 parents, carers, or family members who had accompanied PDHL friends or relatives to NHS appointments. All respondents had used NHS services within the last 24 months, with 10% identifying BSL as their preferred language. Qualitative analysis of the open-ended responses generated three key themes: 1) Accessibility challenges, 2) Impact of communication difficulties across the service pathway, and 3) Lack of consistent, effective deaf-aware communication. Overall, 64.4% of PDHL NHS patients reported missing 50% or more of the important information provided during their NHS appointments, and 32% were satisfied with the communication skills of healthcare staff.

**Conclusion:**

This study presents the largest UK-wide dataset of its kind, and findings highlight the widespread non-compliance with the legally mandated Accessible Information Standards (AIS) within NHS services. The communication barriers identified in this study have significant and long-term implications for the well-being of PDHL patients. Utilising these findings, our working group has developed a set of ‘Recommendations For Change’ to improve deaf awareness and effective communication across the NHS.

## Introduction

Effective communication between clinicians and patients is critical for quality patient care. Inadequate communication can lead to medical errors, compromised patient safety, and reduced trust in healthcare providers [1]. The **“provision of accessible information at every stage of the patient journey”** is a core component of the eight Picker Principles of Person-Centred Care, a framework designed to enhance healthcare quality through the integration of patient experiences and perspectives. These principles have been widely implemented across various healthcare specialities internationally [2–5] and have been adopted within the United Kingdom’s (UK) National Health Service (NHS) to promote patient-centred care and improve health outcomes [6]. The UK’s National Health Service (NHS) is built on principles of equal access and patient-centred care and is legally mandated to meet patients’ communication needs, following the Accessible Information Standard (AIS) [7]. The abbreviation ‘PDHL’ is used throughout this article to describe ‘People who are deaf or have hearing loss’, including those that identify as Deaf, deaf, deafened or hard of hearing. 

Over the last 60 years, extensive research and literature has recognised the communication barriers encountered by people with deafness or hearing loss (PDHL) when accessing healthcare services, globally [1,8–21]. PDHL continue to face substantial barriers in accessing healthcare services, leading to disproportionate and persistent health inequalities. Contributing factors to these inequalities include inadequate access to health information, poor communication, and unnecessary obstacles that prevent patients from receiving care from healthcare professionals [22]. Additionally, PDHL experience disproportionately poorer physical health outcomes [22–24]. Recently, there have been urgent national appeals to improve healthcare accessibility for PDHL in the UK [25–27].

Currently, one in four adults in the United Kingdom (UK) are PDHL (18–80 year olds) [28], and approximately 87,000 of these use British Sign Language (BSL) in England and Wales (likely an underestimate due to insufficient data) [29]. The Royal National Institute for Deaf People (RNID) recently published the “It Does Matter” report which revealed that 24% of individuals with hearing loss, 43% of those who are deaf, and 72% of British Sign Language (BSL) users encountered negative attitudes and behaviours from medical health staff, including audiologists and general practitioners (GPs) [26]. A 2021 survey conducted by a coalition of charities, was completed by over 900 health professionals and NHS patients with disabilities and found that only 11% of patients covered by AIS had equitable access to NHS services [27]. Furthermore, 81% of patients reported that their communication needs were unmet and only 35% of health professionals indicated they had access to AIS-related training through their organisation. Overall, 67% of PDHL reported having no accessible means of contacting their GP. Deaf awareness promotes a better understanding of deafness, as well as the experiences, culture and needs of PDHL [30]. Terry et al. (2024) investigated the experiences of PDHL (n = 66) within the Welsh healthcare context and concluded that further research is necessary to enhance deaf awareness among healthcare professionals [16]. The study emphasises the need for developing and evaluating training resources to improve deaf cultural competencies, with the goal of creating more positive healthcare experiences for PDHL.

This study aims to examine the communication experiences of PDHL, and their communication partners, within NHS healthcare settings in the UK, and assess their potential influence on healthcare use and patient wellbeing. Utilising a cross-sectional, mixed-methods approach, data were collected through an online survey. Survey data are used to generate key recommendations for change to aid communication, improve access to care and reduce healthcare avoidance, and reduce health inequalities for this population.

## Methodology

### Working group

The authors of the present study, led by the British Society of Audiology (BSA), established a multidisciplinary working group in 2021, to include PDHL NHS patients, clinicians, researchers, representatives from the hearing device industry, and hearing loss/deafness charities. The group was formed to address and improve accessibility to healthcare services, with a particular emphasis on the needs of PDHL communities and their families.

### Ethical approval

The Cambridge University Psychology Research Ethics Committee has provided ethical approval for this study (PRE.2021.076). No participant identifiable data were collected. Participants provided their informed consent by completing an online electronic consent form prior to initiating the online questionnaire.

### Study Design

A mixed-methods cross-sectional online survey. Qualitative data are presented following the Reflexive Thematic Analysis Reporting Guidelines (RTARG) [31].

### Survey development

The survey questions were developed by our working group. External audiologists and PDHL reviewed the survey for content and interpretability. Relevant adjustments were made to enhance usability. BSL videos for the information sheet, consent form and all questionnaire items were created by the Cambridge Deaf Association were added to the online survey platform. The survey aimed to investigate the communication experiences of PDHL as well as family members and carers of PDHL, within the NHS. The survey included a combination of closed multiple-choice questions, rating scales, and open-ended questions. The full list of survey questions can be found in the supplementary information. Study data were collected and managed online using the Qualtrics XM platform, hosted by the University of Cambridge [32].

**Fig 1:**
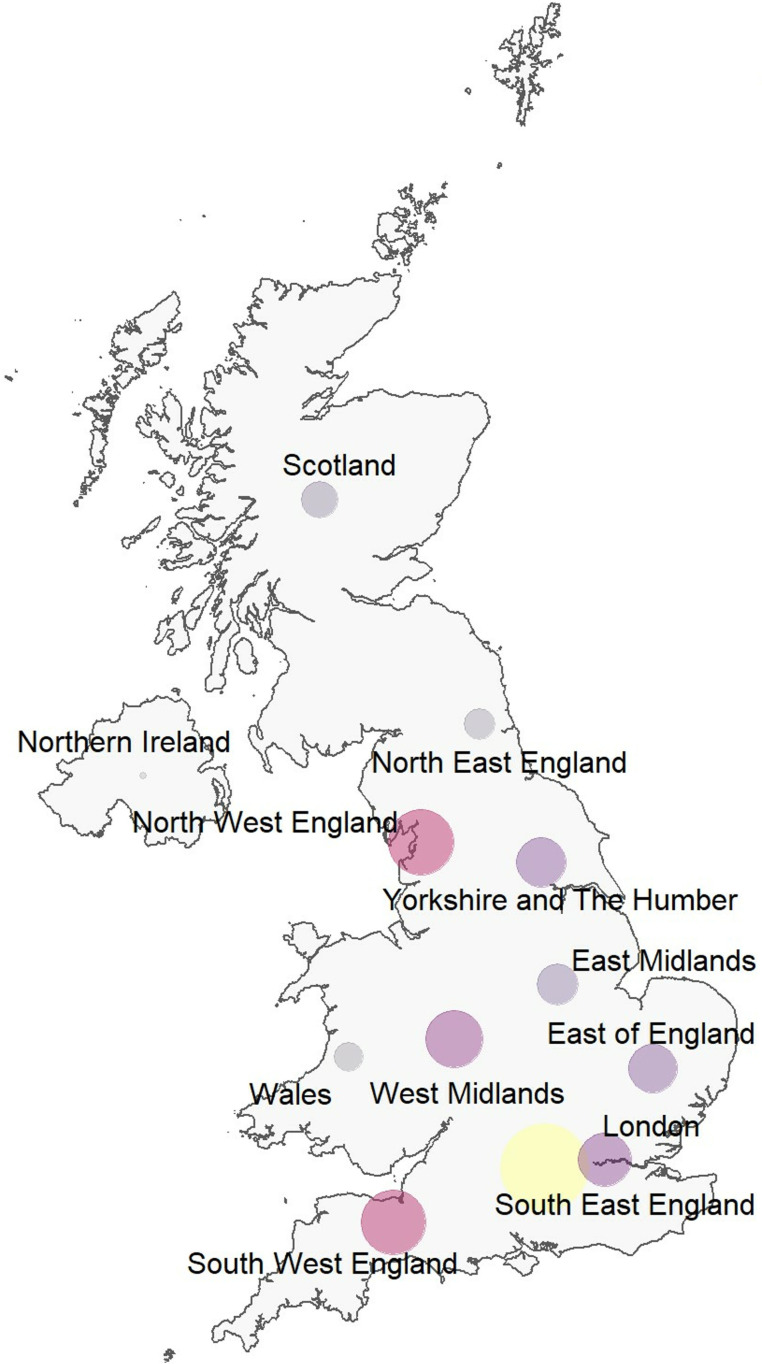
Survey respondents’ regional location.

The survey consisted of the following sections:

1. Rating scales to describe the effectiveness of communication experiences in different NHS settings, a rating scale to identify how much information was missed due to communication barriers in medical consultations and a scale to rate accommodations made to meet communication needs.
2. Open-ended questions were used to explore the impact of communication experiences on emotional well-being, the impact of the COVID-19 pandemic on communication in health services, and suggestions for ways to improve communication and accessibility in the NHS. Open questions were as follows:
  - “Tell us more about your communication experiences”- this question was presented after the rating scales presented in Figure 1
  - “Do you feel the COVID-19 pandemic has changed communication in healthcare settings?”
  - “How do you think we could improve communication with people with hearing loss in healthcare settings?”
  - “What should be prioritised with regards to deaf awareness and communication in the NHS?”
3. Demographic information: Age, sex, ethnicity, regional location, self-reported hearing ability, and use of hearing devices.

### Survey distribution

A link to the online survey was disseminated through social media platforms and distributed via the British Society of Audiology, ENT UK, and RNID platforms, as well as through their membership mailing lists. The survey was available from 7^th^ January to 20^th^ May 2022. Upon accessing the survey link, participants were first presented with an information sheet and consent form, available in both English and BSL.

### Data analysis

Descriptive analyses were used to examine responses to the closed-set questions and rating scales. Reflexive thematic analysis [33] was completed on the open-ended responses following the six-stage approach; [1] Familiarization, [2] Generating initial codes, [3] Generating initial themes [4] Reviewing themes, [5] Refining, defining and naming themes, [6] write up. Given that respondents often provided overlapping answers across different open questions (listed above), the analysis was conducted across all open-ended questions collectively. Two members of the research team independently generated initial codes for the data, with the first author (BP) cross-checking and guiding the resolution of any discrepancies. The themes and subthemes were collaboratively generated initially by the team and subsequently reviewed during an online workshop. This was a flexible iterative process where themes are continuously reviewed, discussed, and refined. The workshop enabled critical reflection, discussion of different perspectives, and ensuring themes capture meaningful patterns in the data.

## Reflexivity statement

Our research team is composed of individuals with diverse expertise in audiology, qualitative and quantitative research, and lived experience of deafness and hearing loss. We acknowledge that our disciplinary backgrounds, professional roles, and personal experiences inevitably shape how we engage with the research process, from data collection to interpretation. Rather than viewing this as a limitation, we embrace reflexivity as an integral part of our approach, recognising that knowledge is co-constructed through interaction between researchers and data. To critically examine our influence on the research, we engaged in ongoing team discussions. These facilitated opportunities to challenge assumptions, explore alternative interpretations, and ensure that our findings remained grounded in participants’ voices. Instead of aiming for consensus, we valued the plurality of perspectives within the team, allowing for a richer and more layered understanding of the data. By embedding reflexivity throughout the research process, we sought to navigate the intersection between our expertise and participants’ experiences, ensuring that our analysis was both informed and critically engaged. This commitment to reflexive practice enhances the depth and integrity of our findings, acknowledging the role of subjectivity in qualitative research.

## Analysis and results

The online survey was completed by 556 people (506 PDHL respondents aged 18–91, mean age: 53.4 years), with 50 parents/carers/family member respondents who had accompanied PDHL friends or family members to attend NHS services. Both groups reported using NHS services in the last 24 months. Overall, 10% (58/556) of respondents reported BSL being their preferred language. Participant demographic data (n = 556) are summarised in Table 1. Figure 1 shows the regional location of respondents.

**Table 1:** Participant demographic information (N = 556). Respondents gave self-reported hearing loss classification of each ear, but classification of the better ear is presented in the table.

Sex	N	%
Female	460	82.7
Male	80	14.4
Non-binary/ third gender	4	0.7
Prefer not to say	12	2.2
Hearing Devices	N	%
Two hearing aids	302	54.3
One hearing aid	52	9.4
One Cochlear implant	58	10.4
One Cochlear implant and one hearing aid	48	8.6
Bone anchored hearing aid	10	1.8
Two cochlear implants	13	2.3
Bone conduction hearing aid	10	1.8
Other	23	4.1
None	40	7.2
Ethnicity	N	%
White British	488	87.8
White – Other White background	14	2.5
Mixed – White and Asian	5	0.9
White – Irish	7	1.3
Black or Black British – Caribbean	1	0.2
Chinese	2	0.4
Mixed – Other mixed background	4	0.7
Mixed – White and Black African	2	0.4
Asian or Asian British – Indian	3	0.5
Asian British other	2	0.4
Asian or Asian British – Pakistani	2	0.4
Other	4	0.7
Prefer not to say	22	4.0
Self-reported hearing loss	N	%
No hearing loss	25	4.5
Mild	36	6.5
Moderate	115	20.7
Severe	165	29.7
Profound	215	38.7

**Fig 2:**
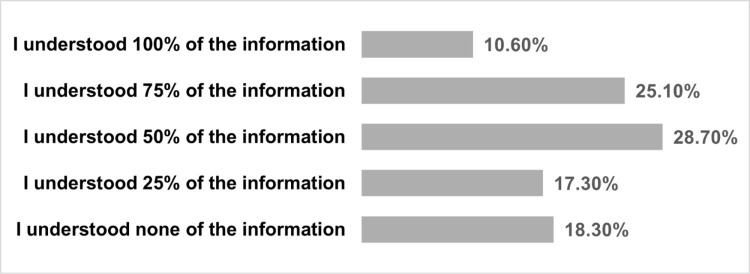
Amount of information understood by PDHL during NHS appointments (N = 506). Respondents were asked whether they feel like they have missed on important information during NHS appointments, because their communication needs were not met.

Figure 2 shows that 10.6% of PDHL NHS patients understood all the important information given during NHS appointments they had attended in the last 24 months, with 28.7% of respondents reporting they understood 50% of the information and 18.3% reporting having not understood any information.

**Fig 3:**
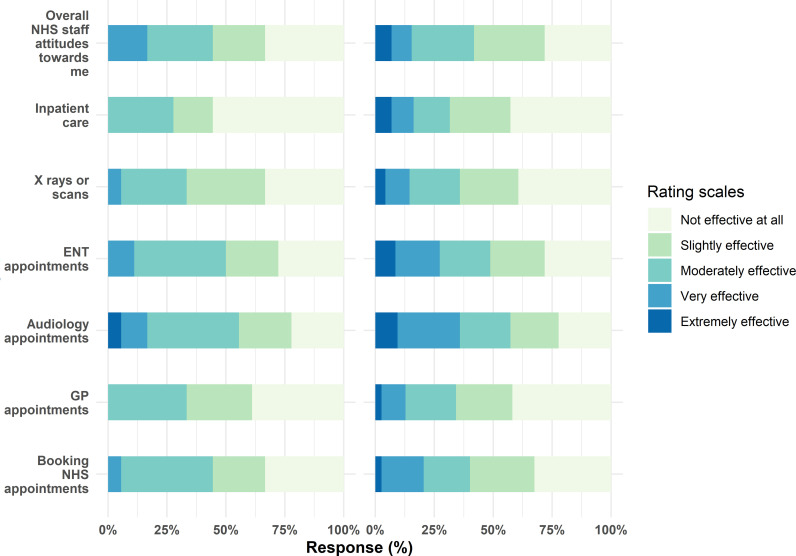
Rating communication experiences in NHS services. Left: Responses from friends/family/carer of PDHL patients (N = 50), Right: Responses from PDHL patients (N = 506).

Respondents rated their communication experiences in a variety of settings including during X-rays or other scans, GP appointments and while booking NHS appointments (Figure 3). They also rated the experiences of audiology and ENT appointments, and overall NHS staff attitudes towards them.

**Fig 4:**
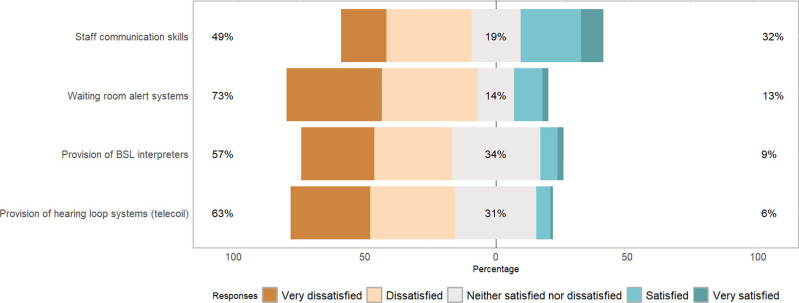
Satisfaction with current accommodations made to optimise communication for PDHL attending NHS services (N = 556).

While rating patient satisfaction of the accommodations made to optimise communication in healthcare settings (Figure 4), 49% of respondents were very dissatisfied or dissatisfied with healthcare staff’s communication skills. Of the respondents who used BSL, 9% were satisfied or very satisfied with the provision of BSL interpreters.

## Qualitative data analysis

The sentiment that *“I always feel like I’m the first deaf person they have ever met”* (83-year-old female, severe hearing loss, no hearing devices) encapsulates the broader experience of PDHL within the NHS. The three key themes are key dimensions of this experience. The main themes are: ‘Accessibility challenges’, ‘Impact of communication challenges across the service pathway’, and ‘Lack of consistent, effective, embedded, deaf aware communication’. An expanded list of quotes from respondents can be found in the supplemental materials.

### Theme 1: Accessibility challenges

This theme discusses the accessibility challenges associated with the inconsistency of care across hospitals and the lack of reasonable adjustments.

#### 1.1 Consistency of care.

Participants describe the lack of uniformity in the support provided to PDHL across various departments. This reflects a broader issue in that many PDHL feel as though their accessibility needs are not anticipated or accommodated. This sentiment captures the experience of many participants who describe inconsistent policies regarding accessibility.

*“I have been told a lot of departments cannot send out emails even though I’m deaf. But [I] never can understand why other departments can.”* (44-year-old female, profound hearing loss, one cochlear implant)

The frustration stemming from the inconsistency leaves patients feeling inferior and frustrated with current systems. Patients often find themselves advocating repeatedly for basic adjustments, reinforcing the feeling that healthcare providers are unprepared to communicate with PDHL.

*“I try not to let it affect me, and I do speak up if I feel I am being treated as ‘less than’ but it can be upsetting…it is frustrating and upsetting when people who should know better do not really have a clue how to communicate with hard of hearing people””* (57-year-old female, moderate hearing loss, hearing aid user)

Participants feel like all medical staff should be deaf aware, so adjustments are made across all settings to accommodate diverse needs.

*“People with hearing loss attend for reasons other than hearing loss and staff awareness of adjustments should be across all settings”* (73-year-old female, moderate hearing loss, hearing aid user).

Furthermore, proper implementation of the Accessibility Information Standard (AIS) could help support effective communication with patients within the hospital setting and prevent PDHL feeling like an anomaly in the system.

*“For a start, the hospital could implement a proper implementation of the AIS - they do nothing at the moment. They need to ensure best practice across the whole hospital with respect to the AIS and things like patient communication packs, not “leave things to individual wards and departments”* (carer/friend/family of a PDHL NHS patient).

#### 1.2 Reasonable adjustments.

There was consistent reference to the lack of reasonable adjustments, meaning that not everyone receives the care they need.

*“The only way to make an appointment is to telephone the surgery to book one. I can’t do this due to my hearing loss. So, I have to visit the surgery in person. There is an intercom to speak to the staff through. I can’t hear over the intercom either. So, I have no way to access my GP”* (51-year-old female, severe hearing loss, hearing aid user)

The inaccessible appointment booking systems due to reliance on telephone communication highlight the necessity for alternative methods such as online booking, email, or text messaging.

*“Sending a text to tell a deaf person to phone them is unhelpful. Giving a number to text back would be helpful”* (47-year-old, severe hearing loss, no hearing device)

These adjustments are reasonable and imperative for ensuring equitable access to healthcare for PDHL.

### Theme 2: Impact of communication challenges across the service pathway

This theme discusses how problems with communication across the service pathway negatively impacts NHS patients and leads to healthcare avoidance.

#### 2.1 Third-party communication.

Many respondents discussed the reliance on family and friends to communicate on their behalf, meaning some people felt they had a childlike dependence on others.

*“I feel absolutely like a child by having to rely in relatives to make calls on my behalf.”* (35-year-old female, severe hearing loss, bilateral hearing aid user).

Due to the necessity of relaying information through a third party, there was a deficiency in maintaining privacy and confidentiality.

*“The importance of booking sign language interpreters and not rely[ing] on family and friends as this is a breach of Article 8 within the Human Rights Act regarding [the] right to privacy and accuracy of relayed information”* (60-year-old male, profound hearing loss, one cochlear implant).

Highlighting the challenges and impacts of depending on others to communicate. Systems need to support people in communicating in a hospital setting in a way that suits their hearing needs and preferred communication methods.

*“Enabling people to communicate in a way that suits them without having to rely on a friend or partner to communicate for them. Treat people as independent individual[s]”* (65-year-old female, severe hearing loss, hearing aid user).

#### 2.2 Advocacy.

Participants describe the exhausting, constant, frustrating battle to advocate about their communication needs, adding stress to an already difficult healthcare experience.

*“Making me feel like I go to war every time I have a medical need”.* (35-year-old female, mild-moderate hearing loss, hearing aid user)

While advocacy is often necessary, it is emotionally and physically draining, particularly for those who are unwell.

*“In general, I am EXTREMELY assertive about my access needs because I know the price I can pay when things go wrong, but doing this asserting is exhausting, and I can’t do it when I’m not mostly-well.* (41-year-old female, severe hearing loss, bone anchored hearing aid user).

PDHL felt that rather than placing the responsibility on patients to repeatedly explain their needs, healthcare systems should be proactive in ensuring consistent accessibility.

*“Why should I have to keep advocating for myself, it should be placed on my NHS record immediately that I prefer BSL support, and this is flagged and arranged for all my appointments or visits without a second thought”* (57-year-old male, profound hearing loss, hearing aid user).

#### 2.3 Impact on emotional well-being.

Across many accounts, participants described the *“”Fear of missing vital information about my health and the consequences there forth”* (36-year-old female, mild hearing loss, hearing aid user). Not being able to hear all of the information can be frustrating and create feelings of anxiety.

*“Humiliated by professionals that don’t speak clearly. It makes me extremely anxious to the point it affects the quality of the appointment”* (35-year-old female, severe hearing loss, hearing aid user).

In some cases, these negative experiences lead to distress long after the appointment.

*“Sometimes I have left appointments upset due to lack of consideration of hearing impairment”* (46-year-old female, mild hearing loss, hearing aid user).

This also led to the feeling that treatment options or onward referrals may not be considered or initiated due to communicational difficulties.

*“I gladly keep good health but fear that I may need emergency assistance and being unable to communicate”* (52-year-old female, severe hearing loss, hearing aid user).

*“I feel very much in limbo, scared and anxious, with no clear pathway for treatment”* (48-year-old female, profound hearing loss, no hearing device).

There are wider implications, as it can also cause emotional strain on family and friends who feel responsible for their loved one’s receiving adequate care.

*“I have found the appointments I have attended with my dad very upsetting. He is not making informed consent to surgical procedures instead relying on me to explain everything after he has signed the consent form. I feel the weight of this responsibility. I worry for those that have no support”* (Carer for 88-year-old male with profound hearing loss).

#### 2.4 Long-term consequences: Healthcare avoidance.

Participants describe that they have experienced negative encounters with healthcare professionals when communication is challenging.

*“Tell them all to stop shouting. I find them all really impatient and aggressive. I dread going to hospital” (*63-year-old female, severe hearing loss, hearing aid user)

The emotional exhaustion of struggling to communicate can lead individuals to disengage from their healthcare.

*“With the GP I find it so difficult and feel so upset by their behaviour that I don’t want to follow up my health needs”* (69-year-old female, profound hearing loss, hearing aid user).

Even healthcare professionals who have hearing loss or deafness themselves acknowledge the bias within the system.

*“Since losing my hearing I feel I am treated less seriously. Despite being a healthcare professional myself the unconscious bias in the medical profession is the first real barrier to overcome before one can even begin to start talking about equal access to health care with hearing loss”* (35 year-old female, moderate hearing loss, hearing aid user)

This has led to individuals delaying or avoiding accessing healthcare altogether, prioritising their emotional well-being over their physical health.

“*It’s demoralising and because of these problems I find GP appointments stressful -each one has been a phone appointment. I delay asking for an appointment until I can’t put it off any longer*.” (62-year-old female, severe hearing loss, hearing aid user)

Accessing healthcare can be a scary experience when someone is living with hearing loss or deafness.

*“The way I have been treated whenever I have needed to attend either a hospital or doctor’s appointment makes me scared to go on my own and I tend to avoid contacting the health services even when it’s likely I need them”* (44-year-old female, moderate hearing loss, hearing aid user).

### Theme 3: Lack of consistent, effective, deaf aware communication

This theme describes inadequate deaf awareness in healthcare.

#### 3.1 Deaf awareness.

Among health professionals, there seems to be insufficient understanding of deaf awareness as “*If staff really understood the issues [deaf awareness], then maybe all the other things would fall into place” (*53-year-old female, severe hearing loss, hearing aid user). Seemingly there needs to be a better understanding of hearing loss and how it can impact patient communication.

*“Get my attention before you speak to me and make sure I can see your face when you speak to me. If everyone did that it would make a huge difference and it is appropriate for everyone not just those with hearing loss”* (72-year-old female, mild hearing loss, hearing aid user).

Healthcare staff would benefit from deaf awareness training. To better understand different communication barriers and make them aware that they may need to employ various strategies to effectively communicate with a range of patients.

*“The issue with general deaf awareness is that it is based on the assumption that a) everyone lipreads to the same level b) all lips are readable c) that BSL (and interpreters) are the only option for a language service professional to support communication between practitioner and patient”* (36-year-old female, profound hearing loss, no hearing devices).

#### 3.2 Variation in care.

There is a variation in whether healthcare professionals adjust their communication style to accommodate PDHL. This lack of consistency means that the quality-of-care patient receives often depends on the individual healthcare provider rather than a standardised, systematic approach.

*“It completely depends on the staff - sometimes they are fantastic and whip out a clear mask so I can lipread, sometimes they carry on mumbling quietly from behind a mask despite me asking them to speak loudly and clearly”* (29-year-old female, moderate hearing loss, no hearing devices).

Staff making an effort to adjust communication is seemingly well received by patients even if it is not always successful; however, communication adjustments should be the norm rather than the exception amongst healthcare professionals.

*“Please be assured I remember the couple of medics who have made an effort to communicate thoughtfully with me, I always express gratitude to such people - despite realising this shouldn’t be exceptional behaviour from “professionals”* (60-year-old male, severe hearing loss, hearing aid user).

#### 3.3 Involving patients and the public in staff development.

Participants expressed that more needs to be done to ensure staff undertake deaf awareness training and that training is developed in coordination with PDHL.

*“Co-produced staff training with people with hearing loss”* (parent of a PDHL NHS patient)

Ensuring relevant and patient-centred training is key to closing the gap in deaf awareness. This includes not just offering training, but also actively monitoring and evaluating its effectiveness.

*“Make sure each NHS hospital/trust has fully implemented the AIS legislation, that this is regularly inspected for compliance with the help of deaf stakeholders”* (carer/friend/family of a PDHL NHS patient).

By integrating direct input from PDHL, hospitals and healthcare providers can move towards a more inclusive system where patients feel expected and accommodated, rather than exceptional or overlooked.

## General discussion

This study represents the first comprehensive investigation of deaf awareness, accessibility, and communication within the NHS, including the perspectives of a large cohort of PDHL, and those who accompany them to healthcare services. The findings reveal that PDHL patients feel that their communication needs are consistently not met within NHS services, resulting in the need to advocate continuously for themselves. PDHL patients are frequently missing critical information at multiple stages of the healthcare journey and the emotional exhaustion of struggling to communicate can lead individuals to disengage from their healthcare services. Furthermore, the existing measures implemented to meet communication needs and comply with legal standards such as AIS, including the use of hearing loop systems, waiting room alert systems, and the provision of BSL interpreters, are not achieving the intended outcomes, because services frequently fail to implement them. Pre-emptive measures are often not taken, meaning that PDHL patients often feel as if their case is exceptional, and their needs are frequently overlooked. This has negatively impacted psychological wellbeing, reduced access to services, and, in some cases, led to healthcare avoidance.

A minority of respondents expressed satisfaction with the provision of British Sign Language (BSL) interpreters. These findings align with research conducted outside the UK, particularly within the US healthcare context, where studies on video remote interpreting (VRI) indicate that, while accessibility to interpreting services has improved with its implementation, satisfaction levels remain inconsistent. The effectiveness of VRI is influenced by several critical factors, including the requirement for high-fidelity video quality and the specialised expertise of interpreters in medical and healthcare contexts [34]. Furthermore, Hommes et al. (2018) investigated sign language interpreters’ perceptions of healthcare communication encounters and recommended the increased implementation of “teach-back” communication strategies to enhance patient understanding [35]. These findings emphasise the necessity of a cohesive approach between interpreting services, healthcare providers, and patients to ensure effective communication. This study also identified limited satisfaction with loop systems (telecoil) in healthcare settings. Research examining the reliability and user satisfaction of loop systems in public spaces within the UK remains scarce. However, existing literature suggests that individuals using hearing devices may not consistently receive adequate information or training regarding additional functionalities, such as telecoil settings, which are crucial for optimising the effectiveness of loop systems [36,37]. Participants in this study reported challenges in accessing general practice services. In 2020, the Royal College of General Practitioners (RCGP) [38], the RNID and NHS England, created the Deafness and Hearing Loss Toolkit to educate GPs and trainees and improve accessibility to services. However, despite these initiatives, persistent difficulties and negative patient experiences suggest that greater awareness and utilisation of these types of resources are necessary. Additionally, systematic monitoring of healthcare facilities, staff training, and adherence to accessibility standards are crucial to improving healthcare access for PDHL patients.

The insights gained from this study can be instrumental in enhancing accessibility infrastructure, developing the healthcare workforce, and ultimately improving the patient experience and health outcomes for PDHL individuals. Our survey findings have informed the development of key recommendations aimed at improving NHS services for **all** patients (see Table 2).

**Table 2.** Recommendations For Change.

Area of activity	Recommendations for change	
**Consistent implementation of existing legislation**	Ensure relevant legislation is implemented consistently and to full effect.	**Collaborate with PDHL, across healthcare development and evaluation, to build trust and share knowledge across communities and healthcare providers.**
**Mandatory training for all healthcare professionals**	Mandatory ongoing deaf awareness training, for all patient-facing NHS staff.	
	Communication awareness training and guidance for clinicians should be co-developed or led by PDHL communities who can share their experiences and perspectives. Training can be digital or in person and informed by themes and subthemes generated in this study, to ensure the diverse range of important aspects of deaf awareness are included. Professionals should use of appropriate personal protective equipment to optimise communication, e.g., clear face masks	
**Appropriate infrastructure**	Develop clinical guidance, policy and infrastructure to create and maintain accessible services, e.g., waiting room alert systems, appointment booking systems that are not reliant on hearing, loop systems, BSL interpreting services	
**Sharing of good practice**	NHS departments should showcase examples of good communication practices to other departments and services Methods of engagement with other disciplines and department, e.g., developing digital strategies with IT and Information governance teams to improve healthcare communication, should be shared.	
**Future research and evaluation**	Large-scale longitudinal review of long-term health impacts of hearing loss and deafness - led by national health bodies Future research examining the impact of communication barriers within healthcare on population-level health disparities Compliance with AIS and other relevant legislation and standards should be monitored and evaluated across NHS patient pathways.	

The NHS long-term plan [39] emphasises the need for a “fundamental shift in how we work alongside patients and individuals to deliver more person-centred care.” For patients to feel both understood by clinicians and to realise the processes and outcomes of clinical consultations, effective patient-clinician communication is essential. This communication is a critical element of person-centred care across all healthcare settings [40–42]. All NHS service providers, along with other publicly funded adult social care services, are legally obligated to comply with the Accessible Information Standard (AIS). This standard applies to individuals who use NHS services and have information or communication needs arising from disability, impairment, or sensory loss. Additionally, the Equality Act (2010) mandates that reasonable adjustments must be provided when necessary, and these adjustments should be implemented proactively rather than reactively. However, the findings of this study, consistent with previous reports [27], indicate that these standards are not consistently upheld within UK healthcare settings, leading to significant negative consequences for PDHL communities, including healthcare avoidance. Healthcare providers and patients should be made explicitly aware of the AIS, particularly given that PDHL may not know their legal entitlements or may lack the confidence to advocate for their communication needs within healthcare settings [15].

Deaf awareness in the NHS is important not only for people who are deaf, but for anyone with diagnosed or undiagnosed hearing loss, which may or may not be disclosed or documented, as well communication challenges arising from other means. It is therefore important to consider individuals’ communication needs regardless of their hearing status within each health and care interaction. Previous literature has reported a lack of deaf awareness in the NHS [16,37,43,44]. Respondents in this study detailed their experiences during NHS clinical consultations, highlighting how the lack of deaf awareness has adversely impacted their access to services, independence, confidence, and psychological well-being. Additionally, they reported feeling a perceived responsibility to self-advocate to secure a better standard of care. They also expressed concerns about the lack of privacy when relying on communication partners to convey medical information. These findings are particularly concerning, as they contribute to healthcare avoidance, which, in turn, is likely to result in poorer health outcomes. Barriers to effective communication and accessibility within healthcare settings seem to be deterring some individuals from seeking timely medical care, exacerbating existing health disparities and negatively impacting overall well-being.

PDHL reported suboptimal communication with clinicians, even within departments specialising in ear and hearing care, such as Audiology and ENT services. These findings are consistent with other studies investigating ear and hearing care services [37,43]. Given that Audiology and ENT departments serve a large proportion of PDHL patients, it is crucial that access to appropriate communication accommodations are fully integrated into these services. There is an opportunity for ENT and Audiology departments, with their extensive experience in working with PDHL patients, to ‘lead by example’ and spearhead improvements in communication practices through collaborative efforts. Efforts have already been made by professional audiology organisations, with new guidance developed as a direct result of this research by the British Society of Audiology [45].

**Fig 5:**
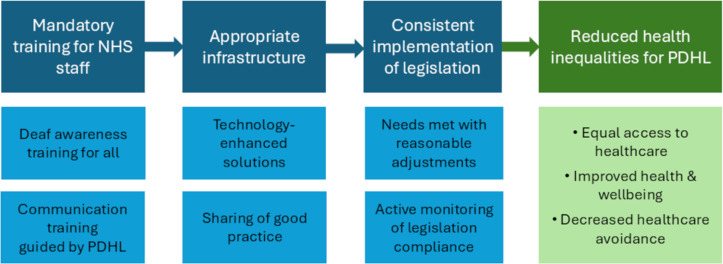
Key Areas and Expected Outcomes of Recommendation Implementation.

Although a principal duty of healthcare professionals is to ‘do no harm’, this undoubtedly focuses on clinical activities and procedures, without acknowledging that poor communication and reduced accessibility to healthcare can have far-reaching and severe consequences, ultimately resulting in harm. Based on this study’s findings, our multidisciplinary working group have developed ‘Recommendations For Change” (Table 2), to improve deaf awareness, communication and accessibility in the NHS. These recommendations should be used to guide health systems to implement both top-down and bottom-up improvements and ensure that the changes are sustainable. Providing reasonable adjustments and following the AIS are legal obligations, and ongoing monitoring of adherence is necessary to identify barriers and facilitators to its implementation. Although this study focussed on exploring the perspectives of PDHL, improving communication in healthcare would be beneficial to all patients and all healthcare services. Therefore, the “**Recommendations For Change**” reported in Table 2 can be applied across healthcare, regardless of patient population or setting. The key areas addressed within these recommendations, along with the anticipated outcomes following their implementation, are presented in Figure 5.

## Strengths and limitations

An important strength of this study is the collaborative development of the survey, which involved input from PDHL, NHS patients and the public, clinicians, researchers, charity representatives. In particular, PDHL contributed to the design of the study, data analysis and writing the manuscript. This is the largest, most comprehensive research exploration of deaf awareness and communication in the NHS, and all regions of the UK are represented. However, the sample predominantly comprised individuals who identified as female and of White British ethnicity, indicating a potential self-selection bias in research participation. This highlights the need for future studies to adopt proactive recruitment strategies to improve representation. The survey was conducted during the national lockdowns imposed by the COVID-19 pandemic. As a result, some respondents specifically noted that the use of face masks exacerbated their communication barriers due to the absence of lip-reading cues. This highlights the need for more accessible personal protective equipment (PPE), such as clear face masks. However, the majority of responses reflected broader healthcare experiences that were not exclusively tied to the context of the pandemic. Therefore, these findings emphasise the ongoing need for inclusive communication in healthcare settings.

## Conclusion and implications

This study presents the largest UK dataset of its kind, and findings highlight the widespread non-compliance with the legally mandated Accessible Information Standards (AIS) within the NHS services. PDHL patients are often unable to effectively access crucial information during healthcare experiences within the NHS. This negatively affects the patient experience, diminishes independence, and may lead to healthcare avoidance. In response, our working group, comprising patients, clinicians, researchers and charity representatives, has utilised the findings from this study to develop recommendations for positive change in this area.

## Patient and public involvement statement

PPIE representatives were involved in the study design, data analysis and writing of this manuscript. They were given training from the research team so they that could carry out qualitative data analysis. Their contribution has been critical to this work.

## Supporting information

S1 File Deaf Awareness Survey. (DOCX)

S2 File Deaf Awareness Dataset. (XLSX)
