# Supplementary material for: “I always feel like I’m the first deaf person they have ever met:” Deaf Awareness, Accessibility and Communication in the United Kingdom’s National Health Service (NHS): How can we do better?
Source: PLoS One. 2025 May 7;20(5):e0322850. doi: 10.1371/journal.pone.0322850 (PMC12057875; doi:10.1371/journal.pone.0322850)
Supplement: S1 File — (DOCX) [file pone.0322850.s001.docx]

**Supplementary materials**

1. Survey questions (excluding demographic questions)

Q1 Are you:

- a person with hearing loss who has used NHS services in the past 24 months? (1)
- a carer / friend / family member who has accompanied a person with hearing loss to use NHS services in the past 24 months? (2)

Q2 How would you rate your overall satisfaction with accommodations made to meet your communication needs in the NHS in the past 24 months?

|  | Very dissatisfied (1) | Dissatisfied (2) | Neither satisfied nor dissatisfied (3) | Satisfied (4) | Very satisfied (5) | N/A (6) |
| --- | --- | --- | --- | --- | --- | --- |
| a. Staff communication skills (1) |  |  |  |  |  |  |
| b. Provision of BSL interpreters (2) |  |  |  |  |  |  |
| c. Waiting room alert systems (3) |  |  |  |  |  |  |
| d. Provision of hearing loop systems (telecoil) (4) |  |  |  |  |  |  |

Q3 Please rate the quality of your communication experiences during the following; (you can give more detail on any of these in the next question)

|  | Not effective at all (83) | Slightly effective (84) | Moderately effective (85) | Very effective (86) | Extremely effective (87) | N/A (88) |
| --- | --- | --- | --- | --- | --- | --- |
| Booking or confirming NHS appointments (1) |  |  |  |  |  |  |
| Communication within GP appointments (2) |  |  |  |  |  |  |
| Communication within audiology appointments (3) |  |  |  |  |  |  |
| Communication within ENT appointments (4) |  |  |  |  |  |  |
| Attending for x-rays or scans (5) |  |  |  |  |  |  |
| Communication during inpatient care (being on a hospital ward) (6) |  |  |  |  |  |  |
| Overall NHS staff attitudes towards me (7) |  |  |  |  |  |  |

Q4 Please use the box below to tell us more about your experiences. Enter as much detail as you wish or type 'N/A' if you do not wish to add more detail.

________________________________________________________________

Q5 How has your psychological wellbeing been affected by these experiences? Or type 'N/A' if you do not wish to add more detail.

________________________________________________________________

Q6 Do you feel the COVID-19 pandemic has changed communication in healthcare settings? If so, please tell us about your experiences, both positive and negative. Enter as much detail as you wish or type 'N/A' if you prefer not to answer.

________________________________________________________________

Q7 Do you ever feel you’ve missed out on important information during NHS appointments, because your communication needs have not been met?

- No, I understood everything (1)
- I understood 75% of the information (2)
- I understood 50% of the information (3)
- I understood 25% of the information (4)
- I understood none of the information (5)

Q8 How do you think we could improve communication with people with hearing loss in healthcare settings? What can we do to make things better? For example: availability of captions, staff training, waiting room visual alert systems Tell us as much as you can, based on your own experience.

Q9 What one thing should we prioritise with regard to deaf awareness and communication within the NHS?

1. Themes, Subthemes and Quotes

| **Main theme** | **Sub theme** | ***Quotes*** |
| --- | --- | --- |
| **Accessibility Challenges** | Consistency of care | *“I have been told a lot of departments cannot send out emails even though I'm deaf. But never can understand why other departments can. It's so stressful trying to sort appointments as they send a letter and can only confirm by phone call or change date via phone cal”l* (44-year-old female, profound hearing loss, CI user)  *“People with hearing loss attend for reasons other than hearing loss and staff awareness of adjustments should be across all settings”* (73-year-old female, moderate hearing loss, hearing aid user)  *“I try not to let it affect me, and I do speak up if I feel I am being treated as 'less than' but it can be upsetting. I try to focus on the positive experiences, but it is frustrating and upsetting when people who should know better do not really have a clue how to communicate with hard of hearing people. I am aware that this is more the fault of hospital management not providing some basic instructions or reasonable adjustments, despite that fact that 1 in 6 people I believe have some form of hearing loss”* (57-year-old female, moderate hearing loss, hearing aid user)  *“For a start, the hospital could implement a proper implementation of the AIS - they do nothing at the moment. They need to ensure best practice across the whole hospital with respect to the AIS and things like patient communication packs, not to leave things to individual wards and departments".* (carer/friend/family of a DHH NHS patient) |
|  | Reasonable adjustments | *“The only way to make an appointment is to telephone the surgery to book one. I can't do this due to my hearing loss. So, I have to visit the surgery in person. There is an intercom to speak to the staff through. I can't hear over the intercom either. So, I have no way to access my GP.”* (51-year-old female, severe hearing loss, hearing aid user)  *“Accessibility to GP services. Impossible to book an emergency appointment at the moment as I cannot hear on the telephone and there is no alternative*.” (44-year-old female, severe hearing loss, hearing aid user)  *“Consistent despair and deaf fatigue having to constantly remind both hospitals and GP practices that there is and has been since August 2016 a legal requirement to meet my communication preferences as given on my records but then no one reading that or responding to what is flagged up”* (63-year-old female, severe hearing loss, hearing aid user) *“Sending a text to tell a deaf person to phone them is unhelpful. Giving a number to text back would be helpful”* (47-year-old, severe hearing loss)  *“Fed up of trying to get [the] GP to understand that I need a video call not a phone call. Fed up of them trying to get me to attend app without interpreter, fed up of them telling to bring a family or friend to interpret for me. Fed up of having to fight constantly for access to the point I would rather prefer not to go to hospital or contact GP. Would rather wait till I am so unwell that it’s an emergency*” (45-year-old female, profound hearing loss, CI user)  *“I was lucky to have a BSL interpreter for every appointment I went so it was good for my well-being. I felt I was highly respected. Without an interpreter I would be anxious and struggling to access important information”* (53-year-old female, profound hearing loss, hearing aid user)  *“I pre research and ask for written materials. In three COVID vaccine appointments only one staff member has acknowledged my need to lipread and removed mask and replaced with clear visor. Any understanding was based on prior research and guesstimates of what they were asking/saying”* (60-year-old male, severe hearing loss, hearing aid user) |
| **Poor Communication challenges across the service pathway** | Privacy/independence | *“I feel absolutely like a child by having to rely in relatives to make calls on my behalf. They have their lives and I feel like I'm a burden to them by asking too many favours.”* (35-year-old female, severe hearing loss, bilateral hearing aid user)  *“I have to be escorted at every appointment as communication is very difficult” (*52-year-old male, severe hearing loss, two hearing aids)  *“Enabling people to communicate in a way that suits them without having to rely on a friend or partner to communicate for them. Treat people as independent individual”* (65-year-old female, severe hearing loss, hearing aid user)  *“The importance of booking sign language interpreters and not rely on family and friends as this is a breach of Article 8 within the Human Rights Act regarding right to privacy and accuracy of relayed information”* (60-year-old male, profound hearing loss, CI user)  *“It would be useful if there were dedicated email/text system solely for deaf patients to use instead of relying on someone else to call for them and losing their independence and privacy” (*30-year-old female, severe hearing loss, hearing aid user).  *“Privacy is important - I talk loud and cannot be expected to hear without being able to read all communication cues - without physical access to medics my right to confidentiality disappears.”* (57-year-old male, severe hearing loss, hearing aid user)  *“...at one point even asking my nine-year-old daughter to confirm something that was my responsibility - not acceptable at all” (*47-year-old female, profound hearing loss, CI user) |
|  | Advocacy | *“In general, I am EXTREMELY assertive about my access needs because I know the price I can pay when things go wrong, but doing this asserting is exhausting and I can't do it when I'm not mostly-well. After my surgery I wasn't in a fit state to fight hard enough for better deaf awareness” (*41-year-old female, severe hearing loss, bone anchored hearing aid user)  *“Start from the premise that the patient is going to struggle to hear and make it a priority to remove that struggle and in so doing remove all the anxiety created by the fear of not hearing and therefore not understanding what is being said. It's vital.” (*67-year-old female, profound hearing loss, hearing aid user)  *“Inclusion that deafness does not mean we can’t do the job, we need to do it differently. We are no less a person because we are deaf*.” (51-year-old female, profound hearing loss, hearing aid user)  *“Why should I have to keep advocating for myself, it should be placed on my NHS record immediately that I prefer BSL support, and this is flagged and arranged for all my appointments or visits without a second thought”* (57-year-old male, profound hearing loss, hearing aid user)  *“Making me feel like I go to war every time I have a medical need”* (35-year-old female, mild-moderate hearing loss, hearing aid user) |
|  | Impact on emotional well-being | *“Humiliated by professionals that don’t speak clearly. It makes me extremely anxious to the point it affects the quality of the appointment”* (35-year-old female, severe hearing loss, hearing aid user)  *“Patience and time are needed to ensure a patient has understood correctly. Very often I feel I am a nuisance or a burden”* (60-year-old female, profound hearing loss, hearing aid user)  *“As a carer I feel ashamed for being hearing and not fighting more”* (a carer/friend/family member who has accompanied a person with hearing loss to use NHS services)  *“Feel totally ignored, invisible and that the health service don’t care at all about accessibility.”* (47-year-old female, profound hearing loss, CI user)  *“Sometimes I have left appointments upset due to lack of consideration of hearing impairment”* (46-year-old female, mild hearing loss, hearing aid user)  *“Loss of confidence, anxiety, fear of communication, fear of missing vital information about my health and the consequences there forth”* (36 year old female, mild hearing loss, hearing aid user)  *“I feel very much in limbo, scared and anxious, with no clear pathway for treatment*” (48-year-old female, profound hearing loss)  *“I have found the appointments I have attended with my dad very upsetting. He is not making informed consent to surgical procedures instead relying on me to explain everything after he has signed the consent form. I feel the weight of this responsibility. I worry for those that have no support”* (Carer for 88-year-old male with profound hearing loss)  *“Psychologically it is difficult to have your needs dismissed by the very service that should understand them. It leads me to believe that I’m a nuisance and they’re not interested in providing a service for people like me”* (42-year-old female, moderate hearing loss, hearing aid user)  *“I have increased anxiety at the prospect of any clinic or GP appt. I gladly keep good health but fear that I may need emergency assistance and being unable to communicate. I'm generally not an anxious person but these thoughts do trouble me” (*52-year-old female, severe hearing loss, hearing aid user) |
|  | Long-term consequences: Healthcare avoidance | *“Tell them all to stop shouting. i find them all really impatient and aggressive. I dread going to hospital” (*63-year-old female, severe hearing loss, hearing aid user)  *“Currently I avoid making routine appointments etc as it is such a stressful situation for me” (*69-year old-female, profound hearing loss, hearing aid user)  *“I now try to avoid contacting the GP if possible”* (60-year-old female, profound hearing loss, hearing aid user)  *“With the GP I find it so difficult and feel so upset by their behaviour that I don’t want to follow up my health needs”* (69-year-old female, profound hearing loss, hearing aid user)  *“I am worried that one day due to lack of accessible appointments I will let something get so bad it kills me”* (41-year-old female, profound hearing loss, hearing aid user)  *“I delay asking for an appointment until I can’t put it off any longer”* (62-year-old female, severe hearing loss, hearing aid user)  *“Fed up of having to fight constantly for access to the point I would rather prefer not to go to hospital or contact GP”* (45-year-old female, profound hearing loss, CI user)  *“The way I have been treated whenever I have needed to attend either a hospital or doctor’s appointment makes me scared to go on my own and I tend to avoid contacting the health services even when it's likely I need them”* (44-year-old female, moderate hearing loss, hearing aid user) |
| **Lack of consistent, effective, deaf aware communication** | Deaf awareness | *“Masks whilst vital during the pandemic, really made communication at appointments hard. You can not see the nurse/doctors lips to fill in the blanks you can not hear. Often at scans the doctor/nurse would talk to you whilst facing an imaging screen and you can not always hear/see what has been said”* (47-year-old female, mild hearing loss, hearing aid user)  *“Get my attention before you speak to me and make sure I can see your face when you speak to me. If everyone did that it would make a huge difference and it is appropriate for everyone not just those with hearing loss”* (72-year-old female, mild hearing loss, hearing aid user)  *“If staff really understood the issues [deaf awareness], then maybe all the other things would fall into place” (53-year-old female, severe hearing loss, hearing aid user)*  *“The notes sent to my GP stated I was a difficult patient to scan as I didn’t breath in when asked. The nurse doing the scan was aware I was deaf but if she just written down a sign for me to breath in it would have been so much better”* (64-year-old female, profound hearing loss, hearing aid user)  *“Wearing my sunflower lanyard makes a big difference to the empath and attitude of staff” (*51-year-old female, profound hearing loss)  *“What would be extremely helpful is including audism* [a negative attitude toward deaf or hard of hearing people] *within deaf awareness training so staff can understand the whole picture and identify ways in which they may be unconsciously hindering communication with patients. The issue with general deaf awareness is that it is based on the assumption that a) everyone lipreads to the same level b) all lips are readable c) that BSL (and interpreters) are the only option for a language service professional to support communication between practitioner and patient*” (36-year-old female, profound hearing loss) |
|  | Variation in care | *“I believe the biggest contribution to help deaf people is people, this can bring the greatest help and support if education is consistent as often new staff, people miss out, it should be available constantly like health and safety*” (72-year-old female, severe hearing loss, bone anchored hearing aid user)  *“It completely depends on the staff - sometimes they are fantastic and whip out a clear mask so I can lipread, sometimes they carry on mumbling quietly from behind a mask despite me asking them to speak loudly and clearly”* (29-year-old female, moderate hearing loss)  *“The staff are generally lovely and very kind and can't fault them, it's the system that's broken. I miss my time slot regularly due to not hearing my name called even after reception telling me they will alert me. Staff no idea about things like live transcribe they could have on tablet to use etc. I have to rely on my Wi-Fi to try to use it so never work in hospital”* (40-year-old female, profound hearing loss, hearing aid user)  *“Please be assured I remember the couple of medics who have made an effort to communicate thoughtfully with me, I always express gratitude to such people - despite realising this shouldn't be exceptional behaviour from "professionals" “*(60-year-old male, severe hearing loss, hearing aid user) |
|  | Involving patients and the public in staff training & service development | *“Input from Deaf people on any changes, or what to change”* (40-year-old, profound hearing loss, hearing aid user)  *“Co-produced staff training with people with hearing loss – work with grassroots communities rather than local governments”*.(parent of a DHH child)  *“employ someone who is Deaf or has hearing loss in the department - that would ease any communication problems”* (51-year-old female, severe hearing loss, hearing aid user)  *“Public commitment and standards that they have to report on. Ideally a deaf engagement group to collate issues”* (60-year-old female, profound hearing loss, hearing aid user)  *“AIS embedded with training given by deaf/ hard hearing”*  (63-year-old female, severe hearing loss, hearing aid user)  *“Make sure each NHS hospital/trust has fully implemented the AIS legislation, that this is regularly inspected for compliance with the help of deaf stakeholders” (*carer/friend/family of a DHH NHS patient) |
